# Community Readiness for Adopting a Physical Activity Program for People With Arthritis in West Virginia

**DOI:** 10.5888/pcd9.110166

**Published:** 2012-03-15

**Authors:** Dina L. Jones, Srilakshmi Settipalli, Jeanne M. Goodman, Jennifer M. Hootman, R. Turner Goins

**Affiliations:** Department of Orthopaedics, West Virginia University; University of Mississippi Medical Center, Jackson, Mississippi; Arthurdale Heritage, Inc, Arthurdale, West Virginia; Centers for Disease Control and Prevention, Atlanta, Georgia; Oregon State University, Corvallis, Oregon

## Abstract

**Introduction:**

The health benefits of physical activity are well established in older adults with arthritis. Despite these benefits, many older adults with arthritis are not active enough to maintain health; therefore, increasing physical activity in adults with arthritis is a public health priority. The purpose of this study was to use the Community Readiness Model to assess readiness for adopting a physical activity program for people with arthritis in 8 counties in West Virginia.

**Methods:**

During 2007 and 2008, we conducted a telephone survey among 94 key informants who could provide insight into their community's efforts to promote physical activity among older adults with arthritis. We matched survey scores with 1 of 9 stages of readiness, ranging from 1 (no awareness) to 9 (high level of community ownership).

**Results:**

The survey placed the counties in stage 3 (vague awareness), indicating recognition of the need for more physical activity programming; community efforts were not focused and leadership was minimal. The interviews suggested that culturally sensitive, well-promoted free or low-cost programs conducted by community volunteers may be keys to success in West Virginia.

**Conclusion:**

Information derived from our survey can be used to match intervention strategies for promoting physical activity among people with arthritis to communities in West Virginia according to their level of readiness.

## Introduction

The health benefits of physical activity are well established in older adults and older adults with arthritis (1,2). Despite these benefits, 53.3% of older adults do not obtain the recommended 150 minutes per week of aerobic leisure activity, and 36.6% do not engage in any leisure-time activity (3). The rate of inactivity is even higher among people with arthritis, even though they can safely engage in regular moderately intense exercise (2,4).

Therefore, promoting physical activity inthe inability of participants to older adults with arthritis is a public health priority (5). This need is apparent in West Virginia, which has the second highest prevalence of arthritis (35% in West Virginia vs 27% in the United States), the third oldest population, and almost one-third of its residents reporting no leisure-time activity (6,7).

From 2009 through 2011, we conducted an evidence-based, community-delivered exercise intervention (EnhanceFitness) at 17 sites (senior centers, churches, recreational, and rehabilitation centers) in West Virginia to determine its effectiveness in people with arthritis. The program includes aerobic, strengthening, flexibility, and balance exercises and improves health-related quality of life and physical function in older adults (8).

Before launching the intervention, we determined the readiness of communities to adopt the intervention after its initial funding ended. The readiness assessment was part of a larger systematic evaluation of the intervention that used the RE-AIM (reach, effectiveness, adoption, implementation, and maintenance) framework (9). The primary measure of adoption is the proportion of organizations that participate in an intervention. Adoption can also involve an assessment of community readiness (10). We used the Community Readiness Model (CRM) to assess readiness and conducted interviews of key informants (11). To our knowledge, only 1 study has applied the CRM to physical activity (12).

## Methods

This cross-sectional study, conducted during 2007 and 2008, surveyed key informants on readiness for physical activity programs for adults with arthritis in West Virginia. We selected 8 counties for the readiness assessment on the basis of their geographical proximity to West Virginia University and our partners at the West Virginia Bureau for Public Health. The study was approved by the West Virginia University institutional review board.

### Community Readiness Model

The CRM was developed for alcohol/drug abuse prevention but has been applied to prevention of HIV/AIDS, domestic violence, and smoking (11,13-15). We chose the CRM because it can be tailored to a particular issue, relies on local experts, and provides individual dimension scores. The CRM has 4 premises: a) communities are at different stages of readiness for addressing an issue; b) the stage of readiness can be accurately assessed; c) communities move through stages to develop, implement, maintain, and improve effective programs; and d) interventions needed to move communities through the stages differ by stage of readiness (16). The CRM includes an interviewer-administered survey for key informants that can be tailored to an issue. The survey has construct validity and high interrater consistency (17).

### Community advisory board

A 14-member community advisory board assisted us in modifying the survey and identifying key informants. The board consisted of representatives of the state health department, the senior services bureau, the Arthritis Foundation, the EnhanceFitness program, and the study sites, as well as community-based participatory researchers, an exercise instructor, and a rheumatologist.

### Key informants

Key informants were community members who could provide insight into their communities' efforts to promote physical activity in adults with arthritis. We considered such people as county health personnel, senior center staff, community leaders, extension service agents, health professionals, social service providers, and rheumatologists.

The CRM suggests interviewing a minimum of 6 key informants per community (18). Our goal was to interview 12 key informants per county (96 people). We assembled a list of informants on the basis of input from the community advisory board, Internet searches, and promotional events at state conferences. We used a snowball technique whereby informants identified other potential informants at the end of their interview. We identified 188 key informants.

### Readiness survey

We tailored the suunity and you know all of your rvey according to procedures in Community Readiness: A Handbook for Successful Change (18). The adapted telephone survey contained 35 questions about community members’ awareness about physical activity in people with arthritis; past, current, and planned physical activity programs; current promotional efforts by community leaders; and potential barriers to program adoption. The survey measured 6 dimensions of readiness (11,16,18,19): a) community efforts, b) community knowledge of efforts, c) leadership, d) community climate, e) community knowledge about the issue, and f) resources. Response options to the questions were in a yes/no format, a numerical rating scale (1 [no awareness] to 10 [very aware]), or open-ended (18). We pilot-tested and revised the instrument using feedback from 5 external reviewers who were representative of the key informants.

Of the 188 recruitment mailings to potential participants, 11 (6%) were returned because of an expired address. Of the remaining 177, fifty-six (32%) could not be reached by telephone or mail, 24 (14%) declined the interview, and 3 (2%) were excluded because they were located outside of the study counties. Overall, 94 of the 177 key informants completed the interview (completion rate, 53%). We interviewed 10 to 15 key informants per county. Respondents and nonrespondents were similar geographically (urban versus rural). There were fewer educators and more health care workers among nonrespondents than among respondents.

### Survey scoring procedures

The interviews were digitally recorded, transcribed for analysis, and scored by 2 independent raters following standardized procedures (18). The interviewer scored each dimension and calculated a stage of readiness score. Each dimension was scored by using an anchored rating scale containing 9 statements ranging from a low (score = 1) to a high level of readiness (score = 9). For example, statements on the anchored rating scale for "Dimension A: Community Efforts" ranged from "no awareness of need for efforts to address the issue" (score = 1) to "evaluation plans routinely used to test effectiveness of many different efforts, results are being used to make changes and improvements" (score = 9).

A second interviewer listened to the audiotape and assigned scores by using the same procedures. The 2 interviewers discussed any discrepancies among their scores until they achieved consensus. We calculated the stage of readiness score by summing the 6 dimension scores and dividing by the number of dimensions. The scores were averaged among informants and then rounded down to the nearest whole number to produce a stage of readiness score. The score was then matched with 1 of 9 stages of readiness in the model ranging from 1 (no awareness) to 9 (high level of community ownership).

### Data analysis

We analyzed categorical data by determining the frequency and proportion for each response item. We deleted missing data in the event of item nonresponse. We calculated median and interquartile ranges for ordinal data. Following procedures described by Plested and Edwards (18), we obtained the mean, standard deviation, and range for each dimension score and the stage of readiness score (18). Responses to open-ended questions were tallied and used to supplement the numerical data. A few informants based their responses on their experiences in more than 1 county, including counties outside the 8-county study area. In such instances, we allocated their responses to the county from which they were recruited for the study. We conducted all analyses using SPSS version 15 (International Business Machines Corp, Armonk, New York).

## Results

The informants were almost equally divided between urban (51%) and rural (49%) counties and represented varied professions; the most common were government (26%), education (20%), health care (19%), and social services (16%). Forty-two percent of informants worked with adults with arthritis and almost all (97%) knew people with arthritis.

### Dimension A: community efforts

Ten percent of informants were aware of arthritis exercise programs that were no longer in existence (Table 1). The reasons cited for stopping these programs included a lack of funding, parking, participants, and instructors.

Thirty-four percent of informants were aware of current exercise programs for people with arthritis. More than one-half of those programs had been active for 5 or fewer years. The median rating for concern of community members about physical activity levels among people with arthritis was 5.0.

### Dimension B: community knowledge of efforts

The median rating for awareness of community members about the existence of programs to promote activity in people with arthritis was 5.0. The most commonly cited strengths of the programs were their free or low-cost nature, the social interactions among participants, and the positive effect of exercise on arthritis symptoms. The most frequently reported weakness was a lack of program marketing.

### Dimension C: leadership

Seventy percent of informants were aware of leaders who were working to promote physical activity in their communities. The types of leaders that were most frequently mentioned by the informants were civic leaders, such as mayors and city council representatives, and local YMCA organizations. Some informants mentioned groups involved with statewide initiatives for promoting physical activity. For instance, 7 informants identified a local researcher who had successfully implemented a physical activity campaign, Wheeling Walks (20).

According to the informants, many leaders were promoting physical activity by developing walking trails and programs. Specific examples of local efforts included the start of a bowling league and a "Biggest Loser" worksite competition, exercise counseling, and incorporation of a popular active video game into schools. The median rating for the perceived concern of community leaders for improving physical activity levels in their community was 8.0.

### Dimension D: community climate

Seventy-one percent of key informants believed that barriers exist. The most commonly cited barriers were a lack of transportation, the inability of participants to afford the cost of attendance, and a lack of interest (Figure). The lack of interest was summed up by 1 informant's comment that "those affected with arthritis do not have a strong voice. There is the attitude that nothing can be done for people who suffer from arthritis." Several informants stated that knowledge was lacking about the existence of programs because of poor marketing. Another informant mentioned that cultural norms were a barrier for people who were not comfortable "exercising in strange groups."

**Figure.**
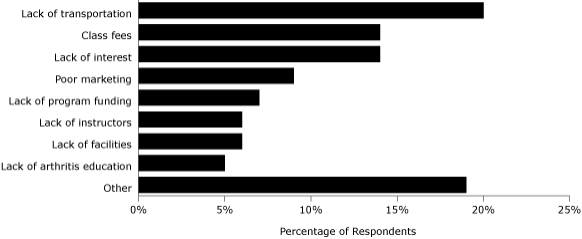
Commonly cited barriers to promoting physical activity among people with arthritis and the percentage of survey participants who identified them. Participants were permitted to identify more than 1 barrier.

**Table:** 

Barrier	Respondents, %
Lack of transportation	20
Class fees	14
Lack of interest	14
Poor marketing	9
Lack of program funding	7
Lack of instructors	6
Lack of facilities	6
Lack of arthritis education	5
Other	19

Programmatic barriers included a lack of site staff and trained professionals to lead the classes and lack of adequate funding. Barriers classified as "other" included factors related to poor infrastructure (location of programs, mountainous terrain for walking, lack of bicycle lanes, inadequate sidewalks), weather concerns, and accessibility for people with disabilities.

Ninety-seven percent of key informants agreed that their community would be supportive in promoting physical activity programs for people with arthritis. When asked why the communities would be supportive, a common theme was community awareness of the need for such programs. Examples of informant quotes to support this theme included the following:

"It's a small community and you know all of your neighbors, so if it was something that would help somebody, people would be interested."

"The average age in West Virginia is increasing and people are becoming more aware, people will be willing to get involved."

"I think there is a genuine need and people want to help."

Only 2 informants indicated that communities would not support programs for people with arthritis. One informant felt that the population of people with arthritis was not "large enough" to gain community support. Another informant thought that community residents had "a natural suspicion of outsiders" and would be less likely to embrace new programs. Despite the barriers, 72% of key informants felt that there was a demand for physical activity programs for people with arthritis, and 86% said there would be interest in new programs.

### Dimension E: knowledge about the issue

The median rating of community members' awareness of the positive benefits of physical activity was 7.0. Forty-five percent of informants thought that information about arthritis was readily available, while only 28% felt that information about the benefits of physical activity was readily available.

The most commonly cited source of information on physical activity was the media (radio, newspaper, and television) followed by hospitals/health clinics, doctors/health care providers, and the Internet. Arthritis information was most likely to be obtained through doctors/health care providers, hospitals/health clinics, the Internet, and senior centers. When asked where a person with arthritis would be most likely to hear about exercise classes, the most frequent response was through the media, followed by health care personnel, senior centers, and word-of-mouth.

### Dimension F: resources

The most commonly cited providers of specialized exercise programs for people with arthritis were the parks and recreation departments, senior centers, churches, community centers, health care facilities, and local extension service offices. Despite the variety of sites offering exercise classes, only 7% of informants were aware of plans to start new exercise programs for people with arthritis. We received few, but positive, responses when we asked about local business' attitudes toward financially supporting those efforts.

Approximately two-thirds of the informants responded that their community had an active volunteer base to help with conducting new exercise classes. Three groups were cited equally as potential volunteers (senior centers, community volunteer organizations, and hospital/medical volunteers). When asked about places in the community that might offer free classroom space for exercise classes, the most commonly identified locations were senior centers, churches, and community centers.

The mean scores for each dimension ranged from 3.1 to 4.5 (Table 2). The overall stage of readiness score was 3.9, identifying the 8 communities as being in the CRM stage of "vague awareness." Stage of readiness scores did not vary by informants' rural or urban location.

## Discussion

The communities' low stage of readiness indicates that the need for more physical activity programming is recognized locally but that current efforts are not focused or detailed, and leadership and motivation are minimal (10). The informants recognized the benefits of exercise for people with arthritis but felt that further education about these benefits was needed. Although few new community-based programs were planned for people with arthritis, the informants felt that their communities would support such programs.

Rural areas face unique challenges in implementing and sustaining public health interventions (21). For instance, rural adults are more sedentary than their urban counterparts and report more barriers to leisure-time physical activity (21-23). The higher prevalence of inactivity in West Virginia could be attributed to the rural built environment (eg, narrower roads, fewer sidewalks), and the low-income and educational levels of the residents (24). Despite these barriers, rural communities have higher rates of readiness for physical activity than suburban and inner-city communities (21-23,25). Readiness in those studies, however, was assessed at the individual level, not at the community level (25). Readiness in this study — with an equal mix of rural and urban communities — was low.

Some of the barriers to physical activity identified in our study, such as a lack of funding and lack of instructors, were found in other studies (26). Other barriers, such as the mountainous terrain or the cultural norm about outsiders, may be unique to West Virginia. Some residents may not feel comfortable exercising in groups with other older adults they do not know. This attitude toward outsiders may not be surprising, given that West Virginia is the second most rural state in the nation and has only 75 people per square mile (27,28). Mistrust of neighbors has been associated with lower activity levels in rural communities (29).

This study had several limitations. Although our unit of measurement was the county, informants identified their community as a county, city, or town. Therefore, informants' responses applied to a range of community sizes. Furthermore, a few informant responses were based on their experiences in more than 1 county, including counties outside the study area.

Although our assessment was conducted in only 8 of West Virginia's 55 counties, it may allow us to generalize our data to the broader state level; the counties in our study are demographically representative of the state's other 47 counties (28). In West Virginia, 49% of the population is male and 95% is white, which is almost identical to the profile of the 8 study counties (29). These counties are also representative of the state on other demographic factors that influence physical activity: the proportion of the population aged older than 65, the proportion of high school graduates, and median household income (28).

According to the CRM, strategies for communities in the vague awareness stage should be aimed at raising awareness, empowering communities to make changes, and soliciting community support (18). Potential strategies could include presenting information at local events about the benefits of physical activity among people with arthritis, the location of current programs, and resources for increasing programming (eg, funding opportunities, instructor training). Other strategies include distributing flyers and posters; creating billboard advertisements; conducting local surveys and sharing survey data with communities; and publicizing physical activity benefits and opportunities through the local news media.

This survey identified barriers and culturally specific issues that could enhance program adoption. Informants suggested that culturally sensitive, well-promoted, and free or low-cost programs may be keys to success in West Virginia. Information derived from our survey can be used to match intervention strategies for promoting physical activity among people with arthritis to communities in West Virginia according to their level of readiness.

## Acknowledgments

We thank our partners at the West Virginia Bureau for Public Health Division of Health Promotion and Chronic Disease, West Virginia Bureau of Senior Services, Southern Ohio Office of the Arthritis Foundation, West Virginia County Aging Providers, EnhanceFitness, and the West Virginia University Prevention Research Center; and Lola Burke, MD, and Anil Kumar Swayampakula, MBBS, MPH, for their technical support.

This project was supported under a cooperative agreement from the Centers for Disease Control and Prevention through the Association of American Medical Colleges grant no. 5U36CD319276, AAMC ID no. MM-0944-06/06.

## Figures and Tables

**Table 1.** Key Informant Responses to a Community Readiness Survey on Physical Activity in Adults with Arthritis in 8 Counties in West Virginia

**Dimension/Interview Question**	No. of Respondents	No. of Responses	Median (Interquartile Range)
**Dimension A: community efforts**			
Were any specialized arthritis exercise programs offered in your community in the last 5 years that are no longer in existence?			
Yes	94	9	NA
No		75	
Do not know		10	
Are there any current specialized arthritis exercise programs or other efforts to promote physical activity among people with arthritis in your community?			
Yes	94	32	NA
No		62	
Do not know		0	
For how many years have these current programs been active?			
≤1	30	5	NA
≤5		12	NA
≤10		7	NA
>10		3	NA
Do not know		3	NA
On a 1 to 10 scale, how concerned is your community about physical activity levels in people with arthritis? (1 = no concern, 10 = very great concern)	94	NA	5.0 (4.0-6.0)
**Dimension B: community knowledge of efforts**			
On a 1 to 10 scale, how aware are people of programs to increase physical activity among people with arthritis? (1 = no awareness, 10 = very aware)	31	NA	5.0 (4.0-7.0)
**Dimension C: leadership**			
Are there any community leaders working to promote physical activity for all community members?			
Yes	94	66	NA
No		28	
Do not know		0	
On a 1 to 10 scale, how concerned are these leaders about improving physical activity levels for all residents in your community? (1 = no concern, 10 = very great concern)	65	NA	8.0 (6.0-8.0)
**Dimension D: community climate**			
Are there barriers in the community to keep people with arthritis from being active?			
Yes	94	67	NA
No		27	
Do not know		0	
Do you think the community would be supportive in promoting physical activity programs for people with arthritis in your community?			
Yes	88	85	NA
No		3	
Do not know		0	
Is there a demand for these programs?			
Yes	57	41	NA
No		11	
Do not know		5	
Do you think that the people with arthritis who you know would be interested in a new exercise class?			
Yes	94	81	NA
No		6	
Do not know		7	
**Dimension E: knowledge about the issue**			
On a 1 to 10 scale, how aware are community members of the positive benefits of physical activity? (1 = not aware, 10 = very aware)	94	NA	7.0 (5.0-8.0)
Is information about arthritis readily available in the community?			
Yes	94	42	NA
No		37	
Do not know		15	
Is information regarding physical activity readily available in the community?			
Yes	94	26	NA
No		60	
Do not know		8	
**Dimension F: resources**			
Are there plans to start new exercise programs for people with arthritis?			
Yes	94	7	NA
No		87	
Do not know		0	
Is there an active volunteer base to help with exercise classes?			
Yes	93	63	NA
No		18	
Do not know		12	

Abbreviation: NA, not applicable.

**Table 2.** Dimension and Stage of Readiness Scores from a Community Readiness Survey of Key Informants (N = 94) on Physical Activity in Adults With Arthritis in 8 Counties in West Virginia

Dimension^a^	Mean (SD)	Range
A: Community efforts	4.0 (2.1)	1.0-7.0
B: Community knowledge of efforts	3.1 (1.3)	1.0-7.0
C: Leadership	4.1 (2.2)	1.0-8.0
D: Community climate	4.5 (1.1)	2.0-7.0
E: Knowledge about the issue	4.1 (0.9)	1.0-6.0
F: Resources	3.8 (0.7)	1.0-6.0
**Stage of readiness score** b	3.9 (1.0)	2.0-6.0

Abbreviation: SD, standard deviation.

a Each dimension was scored using an anchored rating scale containing 9 statements ranging from a low (score = 1) to a high level of readiness (score = 9).

b The Stage of Readiness Score is 1 of 9 stages ranging from 1 (no awareness) to 9 (high level of community ownership).
